# The Pleasurable Urge to Move to Music Through the Lens of Learning Progress

**DOI:** 10.5334/joc.320

**Published:** 2023-09-13

**Authors:** Tomas E. Matthews, Jan Stupacher, Peter Vuust

**Affiliations:** 1 Center for Music in the Brain, Department of Clinical Medicine, Aarhus University Hospital, Nørrebrogade 44, Building 1A, 8000 Aarhus C, Denmark; 2 Royal Academy of Music, Skovgaardsgade 2C, DK-8000 Aarhus C, Denmark

**Keywords:** PLUMM, predictive processing, groove, dopamine, norepinephrine, pleasure, curiosity, learning

## Abstract

Interacting with music is a uniquely pleasurable activity that is ubiquitous across human cultures. Current theories suggest that a prominent driver of musical pleasure responses is the violation and confirmation of temporal predictions. For example, the pleasurable urge to move to music (PLUMM), which is associated with the broader concept of groove, is higher for moderately complex rhythms compared to simple and complex rhythms. This inverted U-shaped relation between PLUMM and rhythmic complexity is thought to result from a balance between predictability and uncertainty. That is, moderately complex rhythms lead to strongly weighted prediction errors which elicit an urge to move to reinforce the predictive model (i.e., the meter). However, the details of these processes and how they bring about positive affective responses are currently underspecified. We propose that the intrinsic motivation for learning progress drives PLUMM and informs the music humans choose to listen to, dance to, and create. Here, learning progress reflects the rate of prediction error minimization over time. Accordingly, reducible prediction errors signal the potential for learning progress, producing a pleasurable, curious state characterized by the mobilization of attentional and memory resources. We discuss this hypothesis in the context of current psychological and neuroscientific research on musical pleasure and PLUMM. We propose a theoretical neuroscientific model focusing on the roles of dopamine and norepinephrine within a feedback loop linking prediction-based learning, curiosity, and memory. This perspective provides testable predictions that will motivate future research to further illuminate the fundamental relation between predictions, movement, and reward.

The affective responses we derive from interacting with art forms such as music, dance, film, and visual art permeate nearly every aspect of our lives, informing our relationships, our identities, and how we spend our time. However, due to their abstract and subjective nature, the psychological and neuroscientific underpinnings of such affective responses have proven difficult to elucidate. A recent and promising approach has been to frame our interactions with such art forms in terms of perceptual learning via probabilistic predictions. That is, such art forms can generate positive affective responses such as pleasure because they provide a means with which to improve the match between our internal models and external input, and thus to generate more accurate predictions in the future (Brielmann & Dayan, 2022; Gold, Pearce, Mas-Herrero, Dagher, & Zatorre, 2019; Mas-Herrero, Maini, Sescousse, & Zatorre, 2021; Van de Cruys, 2017; Vuust, Heggli, Friston, & Kringelbach, 2022). Predictive processes are particularly relevant to music as it is often highly structured in both time and tonal space, unfolding in ways that allow for predictions at multiple timescales (e.g., regarding the next note, the next phrase, the next section). Indeed, many prominent theories of the affective responses to music emphasize the role of prediction violations and confirmations (Belfi & Loui, 2020; Huron, 2006; Koelsch, Vuust, & Friston, 2019; Meyer, 1956; Salimpoor, Zald, Zatorre, Dagher, & McIntosh, 2015; Vuust et al., 2022).

A pervasive phenomenon within both music and aesthetics research is the inverted U-shaped pattern of positive affective responses as a function of stimulus complexity (or familiarity; Berlyne, 1971; Chmiel & Schubert, 2017; Hargreaves & North, 2010). Unlike visual art or film, rhythmic music often elicits a motor response, characterized as the pleasurable urge to move to music (PLUMM) which is associated with the broader concept of groove (Câmara & Danielsen, 2018; Duman, Snape, Toiviainen, & Luck, 2023; Janata, Tomic, & Haberman, 2012; Madison, 2006; Senn et al., 2019; Stupacher, Hove, Novembre, Schütz-Bosbach, & Keller, 2013). PLUMM shows an inverted U-shaped pattern as a function of rhythmic complexity (Matthews et al., 2019; Matthews, Witek, Thibodeau, Vuust, & Penhune, 2022; Sioros, Miron, Davies, Gouyon, & Madison, 2014; Spiech, Sioros, Endestad, Danielsen, & Laeng, 2022; Stupacher et al., 2022; Witek et al., 2014). Predictive processing accounts of this inverted U suggest that medium complexity rhythms achieve the optimal balance between predictability and surprise which results in the greatest PLUMM (Koelsch et al., 2019; Vuust et al., 2022; Vuust, Witek, Dietz, & Kringelbach, 2018; Vuust & Witek, 2014).

However, several questions remain, particularly surrounding the pleasurable component of PLUMM, its relation with the urge to move and other forms of musical pleasure, as well as the role of individual differences in shaping the inverted U. Further, much work on music reward processing has focused on either brief, intensely pleasurable response to music (Grewe, Nagel, Kopiez, & Altenmüller, 2007; Laeng, Eidet, Sulutvedt, & Panksepp, 2016; Martínez-Molina, Mas-Herrero, Rodríguez-Fornells, Zatorre, & Marco-Pallarés, 2016; Salimpoor et al., 2013) or individuals who gain no pleasure from music (Belfi & Loui, 2020; Loui et al., 2017; Martínez-Molina et al., 2016; Mas-Herrero, Zatorre, Rodriguez-Fornells, & Marco-Pallarés, 2014). While studying these extreme cases has provided many useful insights, they do not represent the relatively protracted (i.e., > 1 minute) and moderate intensity responses that likely characterize most individuals’ regular interactions with music.

Here we focus on PLUMM as an illustrative case study, while drawing on research from music reward processing more generally to inform and substantiate our proposal. By drawing primarily on the learning progress hypothesis and integrating it with concepts such as curiosity and creativity, we extend the predictive processing treatment of PLUMM, and music reward processing more generally. We further outline a model of the potential neural mechanisms underlying these processes, focusing on the role of dopamine and norepinephrine in linking the predictive processes to learning, memory, and pleasure.

## Predictive Processing and PLUMM

To elaborate an explanation of the pleasure and motor components of PLUMM and their relation to each other, it is crucial to establish that although they are strongly coupled, they are in fact separable components. Many early studies have focused on the urge to move (Madison, 2006; Madison, Gouyon, & Ullen, 2009; Madison, Gouyon, Ullén, & Hörnström, 2011; Pressing, 2002) while more recent work has shown that this urge is accompanied by positive affect (Janata, et al., 2012; Matthews et al., 2019; Matthews, Witek, Lund, Vuust, & Penhune, 2020; Matthews et al., 2022; Senn, Bechtold, Hoesl, & Kilchenmann, 2019; Senn, Kilchenmann, Bechtold, & Hoesl, 2018; Senn, Rose, et al., 2019; Witek et al., 2014). In these studies, urge to move and pleasure ratings are collected separately and both show an inverted U-shaped function with rhythmic complexity. Although pleasure and urge to move ratings tend to be highly correlated, these correlations tend not to be so high as to be considered colinear, particularly when the ratings are made in two separate listening sessions (e.g., Matthews et al., 2020: *r*(54) = 0.62, 95% CI[0.36, 0.81]). Further, whereas rhythmic complexity affects both components directly, harmonic complexity only affects the urge to move via its effect on pleasure (Matthews et al., 2020). Together these results provide evidence that, although strongly linked, the two components of PLUMM are in fact distinguishable and can be considered separately.

**Figure 1:**
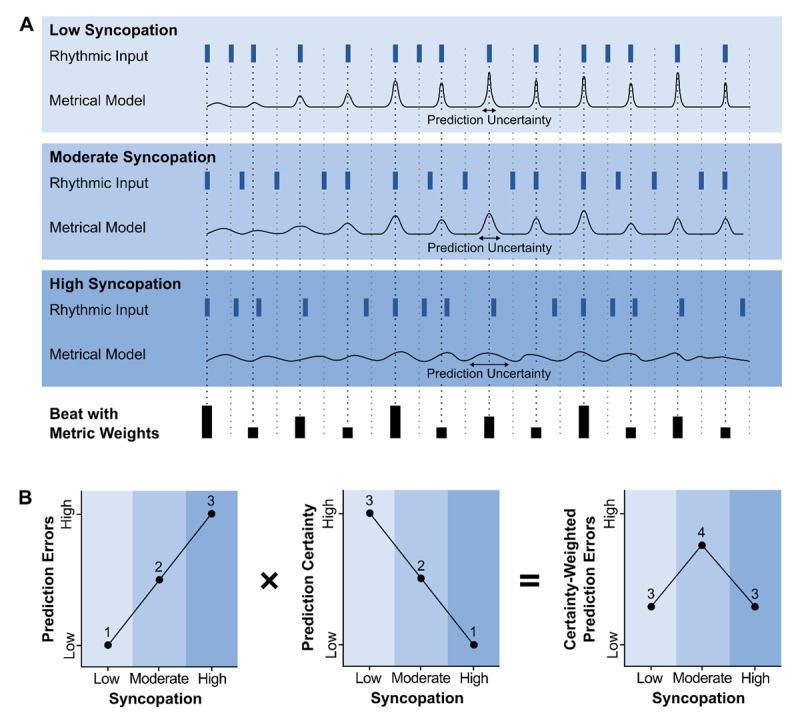
The predictive processing account of PLUMM. **A)** Rhythms with three levels of syncopation lead to meter-based predictions whose uncertainty depend on both the position in the meter and the strength of the metrical model. **B)** Moderately syncopated rhythms maximize the number of strongly weighted prediction errors. Adapted from Stupacher et al., 2022.

Through several influential review and perspective papers, the predictive processing framework, along with active inference, has been deployed to interpret the inverted U-shaped relation between PLUMM and rhythmic complexity (see Figure 1; Koelsch et al., 2019; Vuust et al., 2022, 2018; Vuust & Witek, 2014). The predictive processing framework proposes that the brain uses approximate Bayesian inference to continuously make and update predictions about incoming stimuli and internal states, based on generative internal models, that is, representations of the hidden causes of these stimuli and states (Friston, 2010). Prediction errors, which reflect mismatches between prediction and input, force either a refinement to the internal model or an alteration to the input to better fit the model, e.g., via movement. However, the degree to which prediction errors lead to model improvement depends on the certainty or precision of the antecedent predictions. That is, more precise predictions, if violated, lead to more strongly weighted, or salient, prediction errors, necessitating a stronger model-improving response.

Within this framework, moderately complex rhythms lead to greater PLUMM because they maximize the number of strongly weighted prediction errors. Here, the internal model consists of the beat and meter (henceforth, metrical model). The meter is the pattern of strong and weak beats, including subdivisions, and represents the probability of a note occurring in any position of the metrical grid (Lehrdahl & Jackendoff, 1983; Palmer & Krumhansl, 1990). In Bayesian terms, metrical models reflect context-specific priors that are implicitly learned over years of listening to and/or playing rhythmic music (Jacoby et al., 2021; Kaplan, Cannon, Jamone, & Pearce, 2022). Prediction errors occur when note timings do not conform to the meter, i.e., occur on a position with low probability. For example, syncopations, when notes occur on weak (low probability) metrical positions and are followed by a silence on strong (high probability) metric positions, violate metrical expectations resulting in metrical uncertainty. The degree to which a prediction error affects the metrical model (i.e., its weight), depends on its position in the meter and the strength or certainty of the metrical model itself. Therefore, prediction errors and metrical uncertainty form a feedback loop. As the number of strongly weighted prediction errors increases, metrical uncertainty increases, leading to weaker predictions, indicating the need for a new metrical model (e.g., a different time signature).

Rhythms with a moderate degree of syncopation are predictable enough to allow for relatively strong beat and meter-based predictions, which, when violated elicit strongly weighted prediction errors. However, these strong prediction errors do not invalidate the model but rather indicate the need for modification, resulting in relatively fast and automatic model-updating responses (Lumaca, Haumann, Brattico, Grube, & Vuust, 2019; Vuust et al., 2005). Increasing the number of syncopations can impede the generation of a metrical model, thus predictions are very imprecise, if they can be generated at all. Meanwhile, rhythms with few or no syncopations allow for highly precise predictions, but there are no prediction errors to challenge the model.

Within the predictive processing framework, there are two ways to minimize prediction errors (Friston, 2010); 1) modify the model to better fit the input, e.g., by incorporating syncopations into the model, by phase-shifting the beat and meter to better align with the rhythm (Fitch & Rosenfeld, 2007), or more drastically, by switching to a different meter; 2) change the input, e.g., by moving or tapping along with the purported meter, thus generating additional proprioceptive and sensory inputs that reinforce this meter or satisfy its predictions. Temporal predictions, particularly relative or beat-based predictions, rely on the motor system even when no motor output is necessary or forthcoming (Chen, Penhune, & Zatorre, 2008; Grahn & Brett, 2007; Kung, Chen, Zatorre, & Penhune, 2013; Merchant & Yarrow, 2016; Morillon & Baillet, 2017; Schubotz, 2007; Schubotz, Friederici, & von Cramon, 2000; Teki, Grube, Kumar, & Griffiths, 2011). Therefore, both methods of reducing prediction errors engage the motor system which may underlie the urge to move. Indeed, listening to moderately syncopated, high-groove rhythms, even when not moving, elicits or modulates activity in motor regions in the brain (Matthews et al., 2020; Stupacher et al., 2013). Pleasure is also thought to result from this balance between strong prediction errors and metrical uncertainty (Vuust & Witek, 2014; Vuust et al., 2018), however, this explanation has not been thoroughly elaborated.

Of course, other rhythmic and non-rhythmic features (e.g., microtiming, dynamics, instrumentation, timbre, etc.) can elicit prediction errors and drive affective responses. We focus on syncopations as they are relatively well-studied and show a consistent relation with both perceived complexity (Gómez, Thul, & Toussaint, 2007) and PLUMM (Matthews et al., 2019; Matthews, et al., 2022; Sioros et al., 2014; Stupacher et al., 2022; Witek et al., 2014). It is important to note that defining meter in terms of strong and weak accents or probabilities comes from a western tradition of music analysis. However, similar statistical learning and predictive processes are assumed to form in non-western music traditions (Jacoby & Mcdermott, 2017; Kaplan et al., 2022). Other theoretical models, such as the Dynamic Attending and Neural Resonance theories, have been proposed to account for rhythm and meter perception (Large & Jones, 1999; Large & Snyder, 2009). These accounts also emphasize predictions and prediction errors and therefore do not necessarily conflict with the predictive processing account. Indeed, neural oscillations entrained by the rhythm may provide the neural substrate for the metrical model (Large & Kolen, 1994; Large & Snyder, 2009; Tal et al., 2017). However, the neural mechanisms underlying rhythm perception are beyond the scope of this paper.

## Musical Pleasure

Like primary and secondary rewards, music is a highly motivating stimulus; humans will expend large amounts of time and effort for music or music-related experiences. Although neuroimaging data suggests that they rely on the same brain networks (e.g., Blood & Zatorre, 2001; Blood, Zatorre, Bermudez, & Evans, 1999; Cheung et al., 2019; Gold, Mas-Herrero, Dagher, & Zatorre, 2019; Salimpoor, Benovoy, Larcher, Dagher, & Zatorre, 2011; Shany et al., 2019), aesthetic experiences, such as musical pleasure, are distinct from primary and secondary rewards. Primary rewards, such as food and sex, are directly related to reproduction or the physiological needs that maintain homeostasis. Secondary rewards, such as money, are indirectly related to primary rewards via learned associations (Sescousse, Caldú, Segura, & Dreher, 2013). Therefore, primary and secondary rewards are highly tractable (i.e., more juice or money = more reward). Due to their abstraction and/or distal relation to primary rewards, no such relation exists for music (Hansen, Dietz, & Vuust, 2017). Further, aesthetic experiences are more susceptible to top-down and contextual influences (Brielmann, 2022) as well as interindividual differences, such as cultural background or aesthetic sensitivity (e.g., Clemente et al., 2022; Senn, Bechtold, et al., 2019). Another difference is that primary and secondary rewards are thought of as extrinsic, while musical pleasure is considered to be driven by intrinsic reward processes (Salimpoor et al., 2015). Finally, in computational models of reward-based learning, predictions tend to be about the timing or magnitude of the reward (Schultz, Dayan, & Montague, 1997; Sutton & Barto, 2018). In music, the predictive processes themselves are thought to generate reward (Ferreri et al., 2019; Gold et al., 2019; Hansen et al., 2017; Huron, 2006; Meyer, 1956; Salimpoor et al., 2015; Sloboda, 1991). This highlights that music listening is seen as an active process involving the continuous generation and updating of predictions and that affective responses to music depend not only on the music itself but how we actively engage with it (Mencke, Omigie, Quiroga-Martinez, & Brattico, 2022).

Despite these differences from primary and secondary rewards, prominent theories of affective responses to music emphasize that these responses are rooted in the same fundamental processes as ‘everyday emotions’ such as happiness, sadness, and surprise (Huron, 2006; Juslin, 2013). For example, along with affective responses to music, prediction errors can lead to a fearful startle response or spontaneous laughter at an unexpected punchline (Huron, 2006). It is also important to note that reward, including musical pleasure, is not considered a unitary concept, but consists of three relatively distinct mechanisms (Berridge & Kringelbach, 2015): 1) liking, which refers to the hedonic pleasure of a consummatory experience, 2) wanting, which refers to the motivation to seek out rewarding stimuli, and 3) learning, which is the formation of associations between reward and a given stimuli. One theory suggests that aesthetic experiences reflect liking without wanting, that is, the sensory or consummatory reward mechanisms without the motivational component (Pearce et al., 2016; Scherer, 2004). This applies to aesthetic evaluations, such as beauty or awe, and/or the formation of aesthetic preferences. However, this does not seem to capture the active components of musical pleasure and PLUMM that we are focusing on here, which go beyond, but likely contribute to, ‘mere’ aesthetic evaluations.

Here we propose that many positive affective responses to music, such as PLUMM, are driven by the intrinsic motivation for learning progress (Oudeyer, Kaplan, & Hafner, 2007; Oudeyer et al., 2016; Schmidhuber, 2010). In contrast to the motivation for maximizing extrinsic rewards, intrinsic motivation reflects an internal drive towards activities or stimuli that are themselves enjoyable (Barto & Şimşek, 2005; Ryan & Deci, 2000). This drive may have evolved via its benefit to survival (Singh, Lewis, Barto, & Sorg, 2010). For example, learning to detect and predict regularities in sounds could feasibly impart an evolutionary advantage (Juslin, 2013). Therefore, we may be pre-wired to maximize learning progress, but still must seek out and isolate the stimuli and activities that afford maximal learning by engaging with our environment (Melnikoff, Carlson, & Stillman, 2022). In the context of predictive processing, learning is understood as the improvement of an internal model, that is, increasing the fit between model and input by refining the model or generating new input that satisfies the model’s predictions (Friston, 2010). This reflects perceptual learning that is implicit and automatic, occurring spontaneously as one engages with their environment in intuitive ways without some explicit goal in mind, aside from maximizing pleasure or fun (Schmidhuber, 2010). Note that in this context, we are not referring to the statistical learning that forms or improves higher level schemas (e.g., Loui, 2022). Instead, we are assuming that individuals come in with established metrical models (Kaplan et al., 2022) and that learning reflects continual refinement of these established models to better match the incoming input.

## The Learning Progress Hypothesis

**Figure 2:**
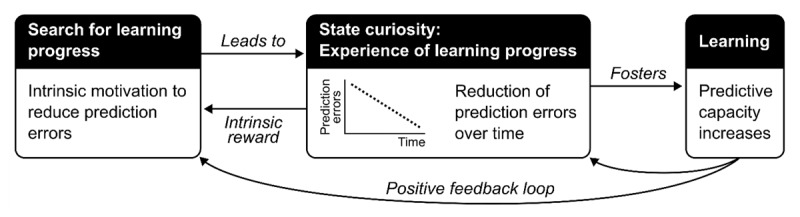
The learning progress hypothesis. Humans are intrinsically motivated for learning progress, which is operationalized as the rate of prediction error minimization over time. The detection of reducible prediction errors mobilizes resources associated with state curiosity to maximally capitalize on the learning potential. Learning progress is registered as pleasure and enhances memory encoding, which in turn facilitates further learning progress, setting up a feedback loop. Adapted from Oudeyer et al., 2016.

According to the learning progress hypothesis (LP), humans are intrinsically motivated to seek out stimuli or activities that maximally afford model improvement (see Figure 2; Oudeyer et al., 2007; Oudeyer et al., 2016; Schmidhuber, 2010). Therefore, given an internal model relevant for a given stimuli space, e.g., relative to similar stimuli within an experimental task, or relative to other music within a given genre, humans will prefer and actively seek out stimuli within this space that optimally challenges this model. ‘Optimal challenge’ here refers to stimuli that are ‘just beyond our predictive capacities’, meaning those that elicit prediction errors but are not so complex as to be unlearnable (Oudeyer et al., 2016, pg., 9). In other words, humans will seek out and preferentially engage with stimuli and activities that engender, not just any strongly weighted prediction errors, but specifically those that are reducible via refinement of the current model. For example, hearing scat singing in the middle of your favourite heavy metal song would result in a large prediction error that would not be easily integrated into your model of heavy metal music or of that particular song, and would thus likely be experienced as aversive. Conversely, hearing scat singing in the middle of your favourite jazz standard may still lead to a prediction error, but this error is more easily reduced by updating your current model of that standard, or jazz in general, and will thus be less likely to cause aversion. Therefore, if we cannot reduce prediction errors by refining our current model, or our current model fails to generate predictions and needs to be abandoned altogether, the stimuli will not afford learning and will be considered unpleasant or boring. Conversely, simple stimuli that align closely to our model will not afford model improvement and thus will also lead to boredom. Accordingly, the learning progress hypothesis predicts an inverted U-shaped relation between complexity and positive affective responses. However, engaging with stimuli with moderate complexity is not the goal in and of itself but an emergent property of the motivation to maximize learning progress (Oudeyer et al., 2016). Further, ‘optimal complexity’ is not fixed but will be individual- and context-specific. Indeed, learning progress itself may transform a stimulus from ‘too complex’ to ‘just right’ over time.

According to LP, the detection of reducible prediction errors, and thus learning potential, leads to the mobilization of resources. This includes increases in arousal, sensory gain, and effort to maximally capitalize on the learning opportunity. An engaged, aroused state enhances the integration of new input and thus the memory of the relevant stimuli or stimulus features. This sets off a positive feedback loop wherein an increase in prediction error minimization promotes further active engagement and motivation to seek out other ‘niches for learning progress’ (see Figure 2; Oudeyer et al., 2016, p. 11). That is, as one learns, their predictive capacity increases, thus continually redefining what constitutes learning and thus the nature of the stimuli that is sought out. For example, participants will attend to more and more complex stimuli as they gain experience with a given task (Forest, Siegelman, & Finn, 2021).

Curiosity is central to the learning progress hypothesis (Oudeyer et al., 2016). A common definition of curiosity is the intrinsic motivation for information gain (Dubey & Griffiths, 2020; Loewenstein, 1994), which highlights its overlap with learning progress. Curiosity can be framed and studied in terms of epistemic (e.g., in trivia paradigms; Kang et al., 2009) or perceptual information gain (e.g., with blurred or partially revealed images; Jepma, Verdonschot, van Steenbergen, Rombouts, & Nieuwenhuis, 2012), with common psychological and neuroscientific mechanisms. In addition, curiosity can be thought of in terms of a trait, that is, a relatively stable part of our personality, or a state, wherein certain situations, environments, or stimuli, temporarily increase the expectation of information gain (i.e., learning). Within LP, the aroused, engaged state associated with detecting learning potential in the form of reducible prediction errors is linked to state curiosity and, within the feedback loop, an increase in the minimization of these prediction errors leads to further state curiosity. The engaged, curious state that accompanies the detection of learning potential is thought to foster better memory retention. This aligns with studies showing greater retention of information that elicit greater curiosity (Marvin & Shohamy, 2016).

Certain theories of curiosity overlap particularly strongly with LP. For example, one theory suggests that curiosity is driven by the urge to maximize the value of one’s current model/knowledge, which can explain why curiosity is triggered by moderately complex stimuli in some situations and novel stimuli in others (Dubey & Griffiths, 2020). Other theories emphasize the role of prediction errors in spurring curiosity, as they indicate a gap between the input and ones current model/knowledge and thus uncertainty about their model/knowledge, motivating further engagement (Gruber & Ranganath, 2019). As in LP, not just any prediction errors will do as curiosity is stronger as participants feel closer to the answer (Wade & Kidd, 2019), that is, when they are in the ‘region of proximal learning’ (Metcalfe, Schwartz, & Eich, 2020). This requires a metacognitive assessment of one’s current knowledge in relation to the input that determines one’s curiosity about the answer to the trivia question or what the rest of the image looks like. Conversely, the curiosity-learning cycle that is relevant in a music listening context is likely to be too fast and automatic to involve such an explicit, metacognitive assessment. However, as we discuss below, moving to music can provide an overt expression of, and thus metacognitive access to, our meter-based predictive processes.

Despite its highly dynamic nature, learning progress can be simply operationalized as the rate of reduction of prediction errors over time; the greater the negative slope of prediction errors over time, the greater the reward (Oudeyer et al., 2016; Schmidhuber, 2010). In a recent study, researchers modeled participants behaviour in a free-choice task using an algorithm that included a linear combination of task performance over all trials and the improvement in the later compared to earlier trials (Ten, Kaushik, Oudeyer, & Gottlieb, 2021). Models with both variables best predicted both participants’ choice of task and time spent on each task. This suggests that participants monitored their learning progress along with overall performance to choose tasks that were not too easy or too complex but provided an optimal challenge. Similarly, Brielmann and Dayan (2022) developed a computational model of the aesthetic value of visual images. This approach involved two generative models, one for predicting the next image (i.e., the immediate sensory environment) and one for predicting likely future images in the long term. Participants’ ratings of images were then simulated based on the degree to which an image aligns with their short-term model (i.e., sensory prediction error) and the degree to which it improves the longer-term model (longer term learning). This model accounted for participants’ ratings, including individual differences, as well as changes in ratings over time (Brielmann, Berentelg, & Dayan, 2023; Brielmann & Dayan, 2022). Importantly, this work highlights the roles of both predictive processing and learning in affective responses to sensory stimuli.

## LP and PLUMM

Within the context of LP, we can reframe the inverted U-shaped relation between degree of syncopation and PLUMM in terms of the reward elicited by an increase in prediction error minimization and the resolution of metrical uncertainty. As described above, moderately syncopated rhythms provide both prediction errors that indicate learning potential and enough regularity to allow for a relatively strong metrical model which can be leveraged to reduce these prediction errors. Accordingly, these rhythms lead to the greatest engagement in terms of both affective and motor responses. Conversely, highly syncopated rhythms elicit so much metrical uncertainty that minimizing or even detecting prediction errors is difficult or impossible. Therefore, there is little or no potential for learning and thus boredom or aversion. For simple rhythms with little or no syncopation, most if not all notes align with the metrical model, therefore there is no prediction errors, no potential for model improvement, again leading to boredom.

The inverted U associated with PLUMM results from operationalizing rhythmic complexity as the weighted sum of its syncopations (Fitch & Rosenfeld, 2007; Longuet-Higgins & Lee, 1984; Witek et al., 2014). However, this measure discounts the influence of individual syncopations and how this influence may depend on, and contribute to, the rhythmic context (Sioros, Madison, Cocharro, Danielsen, & Gouyon, 2022). Due to the relatively fast and dynamic nature of musical rhythms (e.g., compared to harmonic progressions), responses to individual onsets are difficult to measure. One approach is to exploit the high temporal resolution of electroencephalography. For example, one study using this approach shows that fast neural indices of prediction errors are smaller for more complex rhythms (Lumaca et al., 2019), which presumably give rise to weaker metrical models. However, this approach cannot assess how individual notes influence affective responses, how they contribute to modifications to the metrical model, or the relation between these processes. Outside of rhythm, researchers have used a computational model to show that curiosity and pleasure are highest for melodies and chord progressions that balance prediction errors and uncertainty (Cheung et al., 2019; Gold et al., 2019; Omigie & Ricci, 2022). These results suggest that in low uncertainty contexts in which listeners can form strong predictions, opportunities for learning—in the form of prediction errors—are highly salient, leading to greater state curiosity and pleasure. Conversely, when model uncertainty is high, pleasure is driven by predictable notes that resolve uncertainty and thus reinforce the model.

Applying LP to PLUMM highlights that all three components of reward are likely in play while engaging with music. That is, ‘wanting’ can be linked to the motivation to reduce prediction errors and resolve the metrical uncertainty, while ‘liking’ reflects the pleasure resulting from this process. The ‘learning’ component reflects the association formed between the pleasurable state and the rhythm or song that facilitated the learning progress. This ‘learning’ can be framed in terms of means-ends fusion, in which the pleasure associated with learning progress (the end) gets ‘fused’ to the activity of listening to a particular piece of music (the means; Melnikoff, Carlson, & Stillman, 2022; Szumowska & Kruglanski, 2020). This association may also be extended to similar rhythms, songs, or genres, potentially contributing to higher level preferences or schemas. In most studies investigating PLUMM, participants rate their pleasure and urge to move while sitting and not moving. Therefore, the urge to move may be experienced as a (pleasurable) tension (i.e., ‘wanting’; Witek, 2009), driven by metrical uncertainty, and anticipation of the resolution of this uncertainty via overt or covert movement. This is similar to the tension participants experience while waiting for the answer to a trivia question (Kang et al., 2009). This highlights that only reducible prediction errors should elicit PLUMM. The role of synchronous movement in reducing prediction errors, reinforcing the metrical model, and thus reducing metrical uncertainty is a key tenet of the predictive processing account of PLUMM. This has been supported by recent work showing that both PLUMM and PLUMM-related pleasure are increased when tapping one’s foot to the beat (Spiech, Hope, et al., 2022).

In this context, moving to music externalizes our predictive processes allowing for the metacognitive assessment of the gap between our current metrical model and the input, and thus the potential for learning. Linking back to theories of epistemic curiosity (Gruber & Ranganath, 2019; Metcalfe et al., 2020), movements give the listener explicit feedback regarding their knowledge gap and whether they are in a ‘region of proximal learning’. Moving can also expand the representation of the meter and/or draw focus to other aspects of it, thus expanding these ‘regions’. For example, by embodying the beat, e.g., via foot taps, we offload this representation, freeing up attention to other (e.g., faster) metrical levels which can then be embodied by other bodily movements (Burger, Thompson, Luck, Saarikallio, & Toiviainen, 2014; Mårup, Møller, & Vuust, 2022). However, this metacognitive access is limited by the temporal dynamics of our perceptual and motor systems. For example, there is a limit to how fast humans can move, thus limiting our ability to reduce prediction errors at very fast metrical levels (Repp, 2003). In addition, recent theoretical and empirical work suggests that our motor system underlies and constrains the perception of regular auditory input (Morillon, Hackett, Kajikawa, & Schroeder, 2015; Poeppel & Assaneo, 2020), which might limit the granularity of our metrical models. Further, there is a difference between how synchronously we perceive ourselves to be moving to the beat/meter, and how synchronously we are actually moving (Franěk, Radil, Indra, & Lánsky, 1987). A recent study showed that perceived synchrony better predicts ratings of PLUMM than objective measures of synchrony (Matthews et al., 2022), supporting the role of movement-supported metacognitive assessment in PLUMM. This highlights the intrinsic aspect of this affective response, i.e., ‘how much I am enjoying myself’ largely depends on how well I think I am doing.

A key feature of applying LP to PLUMM is that it centers the individual and their interaction with the stimuli, rather than the stimuli itself (Oudeyer et al., 2016). For example, the level of syncopation that will maximally potentiate learning will differ between individuals and between contexts. This also aligns with predictive processing treatments of affective responses to music (Schaefer, Overy, & Nelson, 2013). There is evidence that the shape of the inverted U associated with PLUMM varies according to several inter-individual factors such as musical training, age, and neurological health (Cameron, Caldarone, Psaris, Carrillo, & Trainor, 2023; Matthews et al., 2019; Matthews et al., 2020; Matthews et al., 2022; O’Connell, Nave-blodgett, Wilson, Hannon, & Snyder, 2022; Pando-Naude et al., 2023). According to an LP account of PLUMM, the state of the metrical model should influence the level of rhythmic complexity that will maximally afford learning and thus maximize PLUMM. Extensive musical training may lead to more developed and refined metrical models (Palmer & Krumhansl, 1990; Vuust et al., 2005; Zhao, Gloria Lam, Sohi, & Kuhl, 2017), thus altering the degree of syncopation that will fall just beyond a musicians’ predictive capacity. Conversely, healthy aging and Parkinson’s disease lead to a flattening of the inverted U (Pando-Naude et al., 2023), possibly due to weakening of the metrical model. However, other factors are likely to contribute, including working memory (Vuvan, Simon, Baker, Monzingo, & Elliott, 2020), trait curiosity (Galvan & Omigie, 2022), stimulus familiarity, and preference (Madison & Schiölde, 2017; Senn, Bechtold, et al., 2019).

Memory is a key component of learning, which, in the current context, reflects long term changes to metrical models to better account for expected future rhythms (Brielmann & Dayan, 2022). A recent review suggests that the regularity of musical rhythms and the reward derived from listening to them could improve learning and memory, including for features that are incidental to the rhythm (e.g., speech; Fiveash et al., 2023). Although our proposal suggests a different causal direction, i.e., that learning drives music-induced reward, a key part of LP is that the detection of learning potential mobilizes resources including working memory and long-term encoding (Oudeyer et al., 2016). Outside of music, there is a positive link between curiosity and recall, even for stimulus features that are incidental to the information gain (Gruber & Ranganath, 2019; Kang et al., 2009). Within music, there is evidence of better recall of pleasurable melodies (Ferreri et al., 2021; Ferreri & Rodriguez-Fornells, 2022; Ferreri & Rodriguez-Fornells, 2017) as well as a link between intrinsic motivation for learning and recall (Ripollés et al., 2016). Meanwhile, studies on motor learning using musical sequences suggest a strong connection between motivation, predictability, liking, and learning performance (Bianco, Gold, Johnson, & Penhune, 2019; Fasano et al., 2020). Regular auditory rhythms facilitate perceptual and cognitive performance (Morillon, Schroeder, Wyart, & Arnal, 2016; Stefanics et al., 2010) which support learning, likely via the entrainment of attentional oscillations (Large & Jones, 1999). Whereas adding irregularities may disrupt this process, rhythms that are complex enough to potentiate learning are still regular enough to be accounted for by listeners’ current metrical model. Therefore, these rhythms may balance the regularity necessary for conferring perceptual advantages via entrainment, and the effects of intrinsic learning-based reward on memory processes.

The highly structured nature of music, along with recently developed methods for tracking the complexity of music in a way that aligns with perception (Pearce & Wiggins, 2012; Senn, 2023), makes testing LP within musical contexts not only feasible but highly promising. One approach could be to apply the computational approach of Brielmann et al., (2022; 2023) to rhythmic stimuli. For example, one could simulate affective responses to rhythms of varying complexity based on immediate and rhythm-level prediction errors (e.g., using Bayesian surprisal; Senn, 2023), along with the longer term influence on the metrical model (e.g., using Kullback-Liebler divergence). This would account for both the detection of learning potential of individual syncopations in the short term, as well as the tracking of learning progress in the longer term. Another approach could be to test state curiosity directly following the approach of Omigie and Ricci (2022). For example, participants could be asked to rate their curiosity regarding the way rhythms of variying complexity will unfold. Depending on their musical training and familiarity with the stimuli, participants would be expected to show greater curiosity for moderately syncopated rhythms. These approaches could be combined with neuroimaging, physiological measures, and/or pharmacological interventions to assess the purported neural mechanisms underlying LP within PLUMM (see below).

## The Time-Course of LP in the context of PLUMM

Given the dynamic nature of music, LP and the resulting affective responses, such as PLUMM, can occur on multiple timescales, from onset to onset, to years of listening to the same song (Brattico, Bogert, & Jacobsen, 2013). Within a single musical piece, three forms of prediction-based learning can be considered (Brielmann et al., 2023); 1) each onset elicits a prediction error or confirmation signal depending on its alignment with the metrical model, 2) these signals are integrated over short epochs relevant to the meter (e.g., phrases or repetitions), 3) to improve longer-term, more stable models thus reducing prediction errors encountered in similar rhythms in the future. Therefore, the specific time-course over which learning progress is monitored and leads to affective responses will need to be determined. There is evidence that participants can form accurate aesthetic judgements of music within 500 or 750 ms, however, these initial aesthetic responses are likely based on timbral or harmonic information (Belfi et al., 2018). Conversely, PLUMM relies on temporal processes requiring at least one or two beat cycles and reflects a low-level but protracted affective response rather than an aesthetic judgment.

Brief, more intense responses can also occur, for example resulting from a slow build up and sudden resolution of metrical uncertainty, a common motif in electronic dance music. Alternatively, a relatively low complexity rhythm may initially be misinterpreted with regards to the type (e.g., 3/4 vs 4/4) or phase of the meter. Altering the meter or its phase can then lead to a sudden reduction of prediction errors (Fitch & Rosenfeld, 2007) and thus an increase in pleasure. These examples may correspond to rhythmic versions of an ‘aha moment’ like that seen in epistemic curiosity where providing the answer to trivia question provides a sudden resolution of uncertainty (Gruber & Ranganath, 2019). A similar example is found in atonal music where the initial lack of perceived structure leads to uncertainty and an exploratory mode of listening (Mencke et al., 2022; Mencke, Omigie, Wald-Fuhrmann, & Brattico, 2019). Eventually, the underlying structure is discovered, leading to a sudden reduction in uncertainty and a brief yet strong increase in pleasure (Mencke et al., 2022).

As discussed above, moving to a rhythm provides a way to decrease predictions errors while revealing new avenues to learning progress. In addition, synchronous movements, or those perceived as synchronous, can provide prediction confirmation signals, and thus a fast and salient indication of learning progress. Therefore, through a decrease in prediction errors and an increase in prediction confirmations, synchronous movement can increase pleasure, suggesting a causal directionality. However, the urge to move, framed as the ‘wanting’ component of reward, is itself potentially pleasurable. Further, refinement of the metrical model may be necessary before one has the urge to move. For example, a prerequisite for moving to a rhythm is perceiving a beat. Then, moving to the beat would lead to further pleasure as more uncertainty regarding the metrical model is reduced. This suggests that the two components of PLUMM are likely bidirectional, engaging both ‘wanting’ and ‘liking’ components of reward in repeating and continuously evolving cycles.

Anecdotally, a given piece of music can induce PLUMM even after many years of regular listening. This may result from the ‘learning’ component of reward and means-ends fusion, where a song or rhythm becomes strongly associated with a motor or affective response even as learning progress is exhausted. This component may also account for the fact that our tastes tend to solidify at an early age when many such associations are being formed (Krumhansl & Zupnick, 2013). Music can be thought of as a multidimensional space that can be explored as it unfolds over time. Due to our limited attentional capacities, we may focus on only a subset of this space at a given moment or take a more holistic mode of listening (Brattico, Brattico, & Vuust, 2017). Therefore, repeated listening can continue to uncover new sources of learning progress that are only apparent, or draw interest, as attention moves within this space. This implies that the degree to which a given piece of music affords learning and induces pleasure over repeated listens will depend on its complexity. Outside of PLUMM, there is evidence that more complex melodies lead to a greater increase in liking over repeated listens (Smith & Cuddy, 1986), however, others have shown decreases in liking and/or no dependence on complexity (Gold et al., 2019; Madison & Schiölde, 2017). Further, many songs rated high in PLUMM, such as those from James Brown or The Meters (Janata et al., 2012), can be quite simple in structure and relatively sparse in terms of instrumentation. One possibility is that relatively sparse, repetitive music facilitates a highly detailed metrical model for which relatively small changes can have relatively large impacts. One example is microtiming, in which small deviations from the metrical grid can lead to reducible prediction errors even within a simple repeating pattern. In this way, new learning potential is uncovered as focus shifts to the finer grained details of a rhythmic pattern. Therefore, the effects of familiarity are complex and likely depend on several factors, including the range of complexity, the ecological validity of the stimuli and listening contexts, genre familiarity, and inter-individual differences.

## The Neuroscientific Underpinnings of LP in the Context of PLUMM

**Figure 3:**
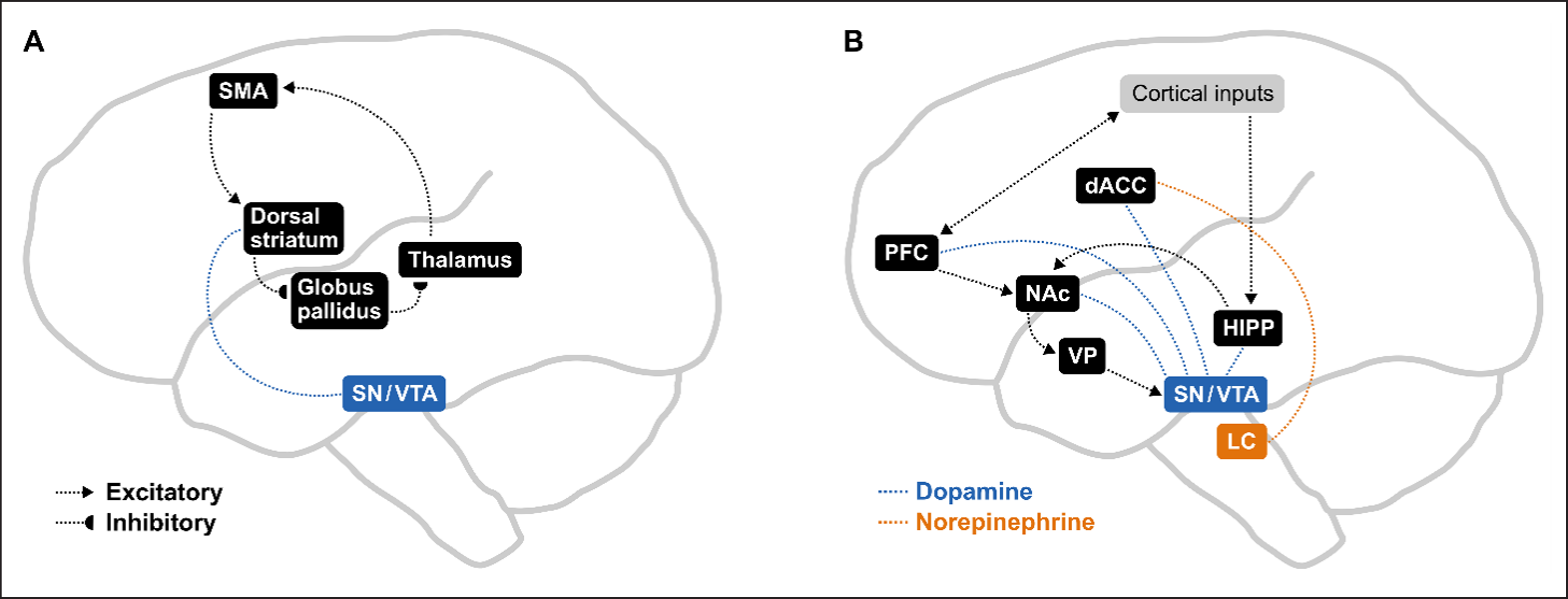
A neuroscientific model of the LP account of PLUMM. **A)** Phasic pulses of nigrostriatal dopamine into the dorsal striatum initiate cycles of meter-based timing mechanisms via excitatory and inhibitory signals within the motor corticostriatal loop. Adapted from Cannon & Patel, 2020. **B)** The detection of reducible prediction errors relative to the metrical model leads to mesolimbic dopamine signals to the hippocampus to enhance memory, and to the dACC which in turn activates the LC to release norepinephrine, leading to the mobilization of attentional resources. The PFC updates metrical models along with higher level schemas. Adapted from Ripollés et al., 2016 and Silvetti et al., 2018. dACC, dorsal anterior cingulate cortex; Hipp, hippocampus; LC, locus coeruleus; NAc, nucleus accumbens; PFC, prefrontal cortex; SMA, supplementary motor area; SN/VTA, substantia nigra/ventral tegmental area; VP, ventral pallidum.

Theoretical and empirical work strongly implicate nigrostriatal dopamine within motor corticostriatal networks as crucial for beat- and meter-based timing (Cameron, Pickett, Earhart, & Grahn, 2016; Cannon & Patel, 2020; Grahn & Brett, 2009). This is in part based on evidence from those with Parkinson’s disease, which is characterized by reduced nigrostriatal dopamine, leading primarily to motor problems, but also changes to cognitive and affective processes (Dauer & Przedborski, 2003). Participants with Parkinson’s show a reduced ability to discriminate rhythms (Cameron et al., 2016; Grahn & Brett, 2009) and judge rhythms as more complex compared to healthy controls (Vikene, Skeie, & Specht, 2019). There is also evidence that musical training counteracts the effect of Parkinson’s on beat perception (Hsu, Ready, & Grahn, 2022). The motor corticostriatal loop, which connects premotor cortical regions and dorsal striatum and overlaps with the nigrostriatal dopamine pathway (Alexander, DeLong, & Strick, 1986), is associated with motor learning (Graybiel & Grafton, 2015). Crucially, the motor corticostriatal loop is strongly associated with processing predictable rhythmic auditory patterns (Bengtsson et al., 2009; Grahn & Brett, 2007, 2009; Grahn & Rowe, 2013; Kung et al., 2013; Matthews et al., 2020; Schubotz et al., 2000; Thaut, 2003). These results, along with the flattening effect of Parkinson’s on the inverted U (Pando-Naude et al., 2023), suggest that the nigrostriatal pathway and motor corticostriatal loop are crucial for the metrical models and predictive processes thought to underlie PLUMM (see Figure 3A).

Along with its role in beat-based timing, dopamine within the mesolimbic pathway is known to play a crucial role in the motivation for primary rewards (i.e., ‘wanting’) and the formation of value-stimulus associations (i.e., ‘learning’; Berridge & Kringelbach, 2015). The mesolimbic pathway involves dopaminergic neurons in the ventral tegmental area projecting to the ventral striatum. The ventral striatum forms the limbic corticostriatal loop with ventromedial prefrontal cortex (Alexander et al., 1986), and is implicated in the experience and anticipation of primary, secondary (Berridge & Kringelbach, 2015; Schultz et al., 1997), and music-induced rewards (Gold et al., 2019; Martinez-Molina, Mas-Herrero, Rodríguez-Fornells, Zatorre, & Marco-Pallarés, 2019; Martínez-Molina et al., 2016; Mas-Herrero, Maini, Sescousse, & Zatorre, 2021; Salimpoor et al., 2011, 2013). Outside of music, mesolimbic dopamine is purported to encode reward prediction errors (RPEs; Schultz, 2016a) which reflect the difference between the expected magnitude of a reward and the actual reward received. In this context, dopamine does not encode the consummatory experience of reward (‘liking’), which is likely controlled by the endogenous opioid system, but is instead involved in the predictive processes necessary for reward-based learning (Berridge & Kringelbach, 2015; Schultz, 2016b).

Recent evidence strongly implicates dopamine in music reward processing. Interestingly, this includes the anticipation of peak pleasurable moments (i.e., chills) during music listening, the motivation to buy preferred music, and the experience of musical pleasure (Ferreri et al., 2019; Salimpoor et al., 2011). This suggests, that unlike primary and secondary rewards, dopamine’s role in musical pleasure includes ‘liking’, along with ‘wanting’ and ‘learning’. Activity in the ventral striatum was also linked to RPEs associated with chord progressions (Gold et al., 2019). However, there is debate regarding how exactly music or musical features can be considered better or worse than expected in terms of (extrinsic) reward (De Fleurian, Harrison, Pearce, & Quiroga-Martinez, 2019; Gold et al., 2019; Hansen et al., 2017). Further, this still leaves open the question of how music-induced reward comes about in the first place.

Along with dopamine’s role in beat-based timing (Cameron et al., 2016; Cannon & Patel, 2020; Grahn & Brett, 2009), the above results suggest that dopamine is crucial to the proposed role of LP in PLUMM. We propose that mesolimbic dopamine signals learning potential via reducible prediction errors, triggering state curiosity, and the associated increase in arousal and attention (see Figure 3B). In this model, nigrostriatal dopamine within the motor corticostriatal loop underlies the metrical model and its relative certainty via phasic pulses and tonic dopamine signals, respectively (Cannon & Patel, 2020; Tomassini, Ruge, Galea, Penny, & Bestmann, 2016). Meanwhile, mesolimbic dopamine within limbic corticostriatal loop detects reducible prediction errors (e.g., syncopations) leading to the mobilization of sensory and cognitive (including memory) resources. This aligns with work implicating dopamine in the link between intrinsic motivation, learning, liking, and memory retention in both language (Ripollés et al., 2018) and melody tasks (Ferreri et al., 2021) via a circuit formed by the ventral tegmental area, the hippocampus, and ventral striatum (Ripollés et al., 2018). In non-human primates, dopamine is modulated by information gain even when this gain involves sacrificing a primary reward (Bromberg-Martin & Hikosaka, 2009). Similarly, in humans, activity in ventral striatum and dopaminergic midbrain increases along with curiosity about the answer to a trivia question, but not when the answer is given (Gruber, Gelman, & Ranganath, 2014). Rather than reward anticipation, this may reflect the reward that accompanies state curiosity. This proposed role of dopamine within LP aligns well with theories linking dopamine to sensory prediction errors and their certainty (Friston et al., 2014; Gershman & Uchida, 2019), rather than RPE’s per se. On the other hand, framing dopamine’s role within LP may provide a bridge between these two hypotheses. That is, since intrinsic reward is linked to learning progress, dopamine may increase along with greater-than-expected learning progress and thus greater-than-expected reward.

The LP hypothesis implies a system in the brain that monitors learning progress derived from a stimulus or activity. For the longer term modification of metrical models, this role may be served by lateral prefrontal cortex (cf. Gruber & Ranganath, 2019). According to a recent model, monitoring learning progress in the shorter term may be driven by the dorsal anterior cingulate cortex (dACC; Silvetti, Vassena, Abrahamse, & Verguts, 2018). The ACC is linked with state curiosity and the mobilization of resources in the face of an information gap (Gruber & Ranganath, 2019). For example, ACC activity is positively associated with perceptual curiosity (Jepma et al., 2012) and melodic prediction errors (Omigie et al., 2019). Further, the ACC is active when listening to rhythms judged as beautiful (Kornysheva, Cramon, Jacobsen, & Schubotz, 2010). The dACC receives dopaminergic input from the ventral tegmental area (Silvetti et al., 2018), which according to our model, signals reducible prediction errors relative to the metrical model (see Figure 3B). At some threshold, dACC signals the locus coeruleus which releases norepinephrine both back to the dACC, and more widely in the cortex. An increase in norepinephrine leads to greater sensory gain, attention, arousal, and increased effort (Mather, Clewett, Sakaki, & Harley, 2016), in other words, leads to the engaged, ready-to-learn state associated with state curiosity.

Norepinephrine is strongly linked with pupil dilation, which has been used as an objective measure of state arousal and effort (Wilhelm, Wilhelm, & Lüdtke, 1999). Recent studies have shown that listening to music with higher PLUMM leads to greater pupil dilation (Bowling, Ancochea, Hove, Fitch, & Madison, 2019). Another study showed greater pupil response for rhythms considered low or medium in PLUMM, particularly when the isochronous hihat was removed (Skaansar, Laeng, & Danielsen, 2019) which may increase reducible metrical uncertainty to further potentiate learning. Finally, pupil dilation as well as drift in pupil dilation over time show an inverted U-shaped function with rhythmic complexity (Spiech, Danielsen, Laeng, & Endestad, 2023; Spiech, Sioros, Endestad, Danielsen, & Laeng, 2022). This pattern of pupil drift was only seen in participants with stronger beat perception, while weaker beat perceivers showed a flattened pupil drift response, supporting the link between strength of the metrical model and state curiosity. In the current context, a stronger metrical model would lead to stronger prediction errors, resulting in greater dopamine signalling to the dACC-locus coeruleus network, greater norepinephrine release, and thus arousal associated with state curiosity. This is further supported by recent work showing greater pupil responses to pitch deviants in more certain melodic contexts (Bianco, Ptasczynski, & Omigie, 2020) as well as a link between pupil dilation and epistemic curiosity (Kang et al., 2009).

## Perspectives on LP, Creativity, Interpersonal Synchrony, and PLUMM

In the following section, we suggest that, in addition to determining the stimuli that humans seek out and enjoy, curiosity and an intrinsic motivation for learning progress may also drive how they create and interact with music (e.g., via dancing). A generative model can only be improved by finding its limits, which can then be expanded as new information is integrated. Accordingly, one role of creative activities may be to self-generate stimuli that challenge our predictive capacities and thus maximally potentiate learning (Schmidhuber, 2010). The classic definition of a creative product has two components; 1. Originality, novelty, or innovation, and 2. Functionality, usefulness, utility, or fit (Runco & Jaeger, 2012). That is, a creative product or act needs to be both novel and useful, however, in more abstract forms of art, such as music, what constitutes utility is less obvious. Within LP, the utility of a creative act or product is the degree to which it affords learning (Schmidhuber, 2010). That is, the novelty or surprise leads to prediction errors, while the utility is determined by the reducibility of the prediction errors. Within PLUMM, this suggests generating rhythms and music that optimally challenge our own meter-based predictions to maximize our own learning progress, pleasure, and fun. As our experience increases and our models improve, it requires more musical skill and knowledge to generate rhythms that provide an optimal challenge. Therefore, in this context, the intrinsic motivation to learn goes hand in hand with the intrinsic motivation for competence and knowledge (Oudeyer & Kaplan, 2007).

The optimal balance between challenge and skill is also a crucial contributor to the psychological construct of flow, which is characterized by a pleasurable, absorptive feeling of high fluency in the context of a relatively difficult task (Csikszentmihalyi, 1990). *Fluency of performance*, a dimension of flow (Engeser & Rheinberg, 2008), was shown to be positively associated with motor synchrony with moderately and highly syncopated rhythms (Stupacher, 2019). This finding suggests that moving to the beat will only induce flow if there is some challenge (Abuhamdeh & Csikszentmihalyi, 2012). A recent model of flow suggests that an activity will induce flow only insofar as it reduces uncertainty regarding the associated goal (Melnikoff et al., 2022), which, in the current context, is learning progress. Therefore, moving to music will lead to flow only if it affords learning, thus helping to narrow which music one will prefer to move to. This can also be linked to music creation in the form of musical improvisation (Norgaard, Bales, & Hansen, 2023; Pressing, 1988) which can be framed as self-reproducing the curious state via the generation of reducible prediction errors. In this context, musical creativity can be seen as a form of exploratory play wherein one can expand their predictive capacities within a uniquely structured and multidimensional space.

This highly structured context also creates an ideal environment for collaborative activities which can further amplify learning progress. Beat and meter allow for a shared, or extended, predictive model across listeners and/or performers thus providing a common structure within which to interact and create (Savage et al., 2021; Stupacher et al., 2022; Vuust et al., 2022; Witek, 2019). Whether dancing or making music, this common metrical model allows for synchronization between individuals as well as with the music, and thus a common space for learning progress where others’ meter-based predictions and prediction errors are communicated via movement. In this context, learning is amplified as people observe the externalization of others’ predictive processes, incorporating and building upon these observations in a cyclic fashion to generate positive feedback loops of learning (Oudeyer et al., 2016). Therefore, a social setting can facilitate collective confirmation and violation of predictions, thus increasing learning progress beyond what would occur alone. For example, prediction errors from a complex rhythm far beyond one’s predictive capacity may still be reduced by observing a more skilled dancer moving to this rhythm (Foster Vander Elst, Vuust, & Kringelbach, 2021). Alternatively, a simple, and thus potentially boring rhythm can be made more interesting as other dancers embellish the rhythm with more complex movements. This suggests that the strong link between interpersonal synchronization and social bonding (reviewed in Fiveash et al., 2023; Savage et al., 2021) may be in part driven by shared learning progress.

Interpersonal rhythmic synchronization has been shown to increase affiliation among participants (Kokal, Engel, Kirschner, & Keysers, 2011), including in young children and even infants (Cirelli, Einarson, & Trainor, 2014; Kirschner & Tomasello, 2009). However, synchronization alone is unlikely to drive the pleasure and affiliation from musical interactions such as dancing at a club or collective improvisation if it does not facilitate learning. To increase learning potential, reducible complexity needs to be injected into the activity. Evidence for this comes from a study using the mirror game paradigm in which dyads try to synchronize their movements but are not instructed with regards to the movements they make. How much the members of the dyad like each other are not only driven by the degree of synchronization but also the degree of complexity of the movements (Ravreby, Shilat, & Yeshurun, 2022). Indeed, participants sacrificed synchrony to increase complexity, suggesting that they prioritized learning and thus the fun of the activity, which in turn increased affiliation. Similarly, in the context of rhythmic music, the perceived affiliation between virtual avatars depends not only on the synchrony with the rhythm but also the complexity of the rhythm (Stupacher, Witek, Vuoskoski, & Vuust, 2020).

## Summary

Here we expand on predictive processing accounts to suggest that the intrinsic motivation for learning progress is a crucial driver of PLUMM, providing a thorough and testable explanation of this powerful and ubiquitous affective response to music. Crucially, this proposal accounts for inter-individual and contextual influences on the inverted U-shaped relation between PLUMM and rhythmic complexity. In addition, this proposal ties together prominent psychological and neuroscientific theories of reward, motivation, and learning, with prediction as a fundamental underlying principle. We suggest that the feedback loop linking learning progress, PLUMM, and memory retention is subserved by dopaminergic and noradrenergic transmission within loops connecting cortical, subcortical, and brainstem regions. This forms the link between neural mechanisms underlying motor and reward processing, which together motivate active, exploratory learning, and creative social interactions. The highly structured nature of music provides an ideal testbed for individuals and groups to test and refine their predictive processes and thus generate learning progress. Further, this structured, and thus tractable, nature makes rhythmic music highly amenable for the investigation of the link between curiosity, learning progress, aesthetic pleasure, and creativity. Along with new technological developments and approaches, these investigations can provide exciting possibilities for researchers to better understand the important role of music in our lives as well as its utility in clinical settings.
